# First evidence of keratinolytic activity in *Simplicillium aogashimaense* isolated from wool-pellet–amended soil

**DOI:** 10.1186/s12934-026-03066-y

**Published:** 2026-07-15

**Authors:** Clémentine Isembart, Zoé Bruchon, Jean-Michel Yao, Boris Zimmermann, Cristian Bolaño Losada, Achim Kohler, Paige Heavyside, Volha Shapaval

**Affiliations:** 1 Faculty of Science and Technology, Norwegian University of Life Sciences, 1432 Ås, Norway; 2 Faculty of Science and Technology, Université de Lorraine, 1bd Aiguillettes, Vandoeuvre les Nancy, 54000 Nancy, France; 3 Woolero AS, Høgskoleringen 4, ved VM-paviliongen, 7034 Trondheim, Norway

**Keywords:** Keratin degradation, Fungi, Bioconversion, Circular economy

## Abstract

**Background:**

Keratin-rich waste such as wool and feather wastes are generated in large quantities worldwide and remain difficult to valorize due to the structural resilience of keratin, that is reinforced by disulfide bonds. Microbial degradation offers a sustainable approach for converting these wastes into value-added products. In this study, keratin-degrading fungi were isolated from garden soils amended with wool pellets used as organic fertilizer. Three fungal strains showing keratinolytic activity were selected based on halo formation on keratin-containing agar media. Among them, *Simplicillium aogashimaense* was chosen for detailed characterization due to its non-pathogenic status, limited representation in the literature, and the absence of previously reported proteolytic or keratinolytic activity.

**Results:**

Feather meal degradation by *S. aogashimaense* was investigated during 12 days of submerged cultivation. The strain demonstrated strong keratinolytic potential, achieving up to 80% feather meal mass reduction. Biochemical analysis of the resulting hydrolysates, including protease activity, elemental composition, amino acid profiles, ammonium and sulfur concentrations, pH dynamics, and overall biochemical profile, revealed a multiphasic degradation pattern. The initial phase (days 1–3) was characterized by rapid feather breakdown, elevated protease activity, ammonium release, and preferential liberation of surface-accessible amino acids, indicating efficient enzymatic attack on exposed keratin domains. The intermediate phase (days 4–6) during which general protease activity declines along with the fungal biomass. This period is likely to correspond to a shift from feather degradation to assimilation and intracellular metabolism. During the final phase (days 7–12), measured parameters stabilized, consistent with maximal substrate degradation and subsequent assimilation of soluble hydrolysis products by the fungus.

**Conclusions:**

This study provides the first evidence that *S. aogashimaense* exhibits keratin-degrading capability, identifying it as a previously unrecognized keratinolytic fungal species. These findings broaden current understanding of fungal keratin degradation and highlight the potential of *S. aogashimaense* for sustainable bioconversion of feather waste and other keratin-rich residues. Further optimization and mechanistic investigation of its enzymatic system may support future biotechnological applications in waste valorization.

**Supplementary Information:**

The online version contains supplementary material available at https://doi.org/10.1186/s12934-026-03066-y.

## Introduction

Annually, billions of tons of keratin-rich wastes are generated from various industrial sectors [3]. Due to a lack of efficient recycling strategies, most of these waste materials are incinerated or landfilled, adding up to the already existing environmental concerns [10]. Keratin-rich materials are characterized by an extraordinary resilience due to the nature of keratin which is structural protein characterized by its α-helical and β-pleated structures, strengthened by disulfide bonds, that provides durability and hinder natural breakdown [14, 15, 16].

Keratinous waste streams are generated primarily by the agro-industrial sector and include materials such as pig bristles, hooves, wool, and feathers. These wastes are produced in large quantities and are rich in nitrogen, making them attractive targets for recycling and valorization. Microbial hydrolysis represents a particularly promising biotechnological approach, as certain bacterial and fungal species secrete keratin-degrading enzymes that convert insoluble keratin into soluble hydrolysates rich in peptides and amino acids [1, 22].

Wool and feathers are both keratinous residual materials; however, their structural properties differ substantially. Wool contains higher proportion of α-keratin, the helix form of keratin secondary structure, similarly to all mammalian keratin rich tissues, whereas feathers contain mostly β-keratin, its sheet form, which is more abundant in non-mammalian keratinous tissues [16]. This difference in composition gives rise to distinct properties: wool’s keratin fibers are more heavily crosslinked due to a higher proportion of disulfide bonds between the keratin chains [32]. This contributes to wool’s rigidity and its increased resistance to degradation. As a result, microbial access to the inner core of wool fibers is more restricted than in feathers. Keratin degradation therefore relies on specialized mechanisms, typically involving keratinases, proteases, and reducing agents, whose effectiveness varies depending on the keratin source [1, 47]. Consequently, the valorization of feather and wool waste requires distinct bioconversion strategies, despite partial overlapping in the mechanisms involved. Moreover, efficient wool degradation has more frequently been reported for fungal strains [17, 37, 39, 41], whereas bacterial systems are more commonly described in the context of feather degradation [7, 24, 29, 43].

Over the past decade, studies have expanded the catalog of keratin-degrading microorganisms, with numerous newly isolated strains reported from diverse environments [2, 4, 37, 43, 49]. All the identified microbes associated with keratin degradation are either bacteria or fungi. Furthermore, dermatophytes account for most of the fungal species. Indeed, traditionally, isolation efforts have targeted environments closely associated with keratin-rich waste, such as poultry farm soils or directly from bird feathers. In contrast, bioprospecting approaches aim to identify novel keratin degraders from less obvious but ecologically relevant niches, such as Antarctic green snow, whose occurrence coincides with penguin colonies and associated keratin inputs [43, 44].

In this study, novel keratin-degrading microorganisms were isolated from garden soil samples amended with Woolero wool pellets (Woolero AS, Norway). These pellets have previously been shown to enhance crop growth, suggesting that microbial communities capable of degrading wool fibers contribute to nutrient enrichment of the soil [55]. The wool-amended soils were inoculated onto keratin-rich agar plates to identify potential keratin degraders, as indicated by the formation of degradation clear zones surrounding microbial colonies. Among the efficient keratin-degrading isolates obtained, *Simplicillium aogashimaense* was selected for further characterization due to its non-pathogenic status, its limited representation in the literature [21], and the absence of any previously reported proteolytic or keratinolytic activity, highlighting its potential novelty. The strain was characterized with respect to its feather meal degradation efficiency, proteolytic activity, and the chemical composition of the resulting hydrolysates.

This work provides the first evidence of keratin degradation and proteolytic potential in *S. aogashimaense*, establishing this fungus as a novel and efficient keratin-degrading microorganism.

## Materials and methods

### Isolation and identification of keratin-degrading fungi from soil samples

#### Soil samples

Soil samples were obtained from an external experiment designed to assess the effect of wool pellets (Woolero AS, Trondheim, Norway) on lettuce growth. In this experiment, 51 treatment conditions were tested, involving four substrate types (garden soil, compost, peat and sand) supplemented with wool (either in pelletized or shredded form) at different application rates and combined with other organic materials (bone meal, eggshells, wine-making mass and potato peals). Each type of soil also presented a positive control (soil amended with a mineral fertilizer), and negative control (soil without wool or fertilizer). Soil samples were collected after the lettuce harvest, and an additional sample of each soil type was collected prior to any amendment and before planting. The compositions of the 51 tested soil samples are reported in Supplementary Table S1.

#### Isolation of potential keratin degrading fungal colonies

To prepare samples for plating, 2 g of soil sample was suspended in 2 mL of sterile phosphate-buffered saline (PBS). The suspensions were then incubated at room temperature on a MaxQ 4000 orbital shaker (Thermo Fisher Scientific, USA) at 200 rpm for 2 h. Following sedimentation, 10 µL of the supernatant was collected and diluted to 1:100 and 1:1000. Two keratin sources were tested in this study: (1) wool, predominantly composed of α-helix keratin and (2) feathers, primarily containing β-sheet keratin. To enable homogeneity into agar media, both materials were processed into meals following the method described by [37]. The resulting wool meal (WM) and feather meal (FM) were stored dry at room temperature and subsequently used to prepare wool-meal agar (WMA) and feather-meal agar (FMA) plates. The basal medium contained (g/L): MgSO_4_ × 7H_2_O – 2; KH_2_PO_4_–0.1; FeSO_4_ × 7H_2_O – 0.01; CaCl_2_ × 2H_2_O– 0.5; agar – 15, and either WM or FM – 10. Chloramphenicol (0.04 g/L) was added to inhibit bacterial growth, as recommended by [25]. 100 µL of the prepared soil extracted solution was spread onto the prepared keratin agar plates using sterile glass spreaders. The plates were then incubated at 25 °C and were inspected daily for a period of 10 days. Colonies exhibiting keratin degradation, indicated by the appearance of a clear halo surrounding a colony, were transferred to fresh FMA and WMA plates for obtaining pure culture of keratin degrading isolates.

Coomassie Brilliant Blue (CBB) staining was used to enhance visualization of keratin degradation halos around colonies grown on WMA and FMA plates. Two solutions were prepared: a washing solution composed of methanol, acetic acid, and water (50:10:40 v/v/v), and a staining solution prepared by dissolving 2.5 g of CBB R-250 powder (Thermo Scientific USA) in 1 L of the washing solution. For staining, 10 mL of the CBB solution was added to each plate and incubated for 2 h at room temperature. After incubation, the staining solution was discarded and plates were washed three times, for 1 h each, using the washing solution to remove excess of dye and reveal clear halos around colonies.

Isolates that consistently demonstrated keratin-degrading activity were subsequently inoculated on malt extract agar (MEA) and incubated at 25 °C until sporulation. For long-term preservation, 10 mL of sterile saline solution was added to sporulating MEA cultures to resuspend spores. A total of 750 µL of the suspension was transferred to a cryotube and mixed with 750 µL of sterile 50% glycerol. The cryotubes were stored at − 80 °C.

#### Identification of the fungal isolates

The identification of the isolated fungi was performed by Westerdijk Fungal Biodiversity Institute. Upon arrival, isolated were cultivated on MEA and dicloran-18% glycerol agar (DG18). DNA was extracted from 3-day-old MEA cultures grown in the dark at 25 °C using the Qiagen DNeasy Ultraclean™ Microbial DNA Isolation Kit. For *Simplicillium* isolates, the internal transcribed spacer (ITS1–ITS2) including the 5.8 S rDNA, and a partial β-tubulin gene (BenA) were amplified and sequenced using primers LS266 and V9G (ITS) and BT2a/BT2b (BenA). For *Penicillium* isolates, partial BenA and calmodulin (CaM) gene regions were amplified and sequenced using BT2a/BT2b and CMD5/CMD6 primers, respectively. The PCR products were sequenced in both directions using the same primers and the BrilliantDye v. 3.1 Cycle Sequencing Kit (NimaGen, Nijmegen, The Netherlands). Sequencing was performed on an ABI PRISM 3700 Genetic Analyzer. Forward and reverse sequences were assembled into contigs using SeqMan (LaserGene package), and resulting sequences were compared against NCBI GenBank (BLAST) and the Westerdijk in-house reference database for species assignment.

### Characterization of the selected fungal strain for keratin degradation

#### Shake flask feather hydrolysis assay

A shake-flask experiment was performed to assess feather hydrolysis by the selected fungal strains. A mineral medium was prepared with the following composition (g/L): MgSO_4_ × 7H_2_O – 2; KH_2_PO_4_–0.1; FeSO_4_ × 7H_2_O – 0.01; CaCl_2_ × 2H_2_O – 0.13. 75 mL of mineral medium was dispensed into 250 mL Erlenmeyer flasks and feather meal was added at a final concentration of 10 g/L and autoclaved at 121 °C for 15 min. Each flask corresponded to a biologically independent sample. Samples were collected at nine time points (days 1, 2, 3 ,4 5, 6, 8, 10 and 12) with two replicates for each one.

Fungal spore inoculum was prepared from 10-day-old MEA cultures grown at 25 °C. Spores were resuspended in sterile water and counted using a Countess™ 3 automated cell counter (Thermo Fisher Scientific, USA). Flasks were inoculated to a final concentration 1 × 10⁵ spore/mL and incubated at 25 °C in a MaxQ 4000 orbital shaker (Thermo Scientific, USA) at 150 rpm (1.9 cm orbit) until sampling. At each sampling time point, the entire content of each flask was centrifuged at 5000 rpm for 10 min at 4 °C. The supernatants (hydrolysate) were separated and stored at − 20 °C until further analysis. The pellets were washed three times with distilled water, freeze dried with a Labconco FreeZone2 2.5 L (LabConco, USA), weighted and store at − 20 °C until further analysis.

#### Mass estimation of soluble and insoluble solids, fungal biomass, and keratin degradation

Aliquots of hydrolysates were accurately measured by volume and freeze-dried to estimate the total soluble solids. This mass contained solubilized keratinous material and salts. The pellet samples weight was used to determine the total insoluble solids, which consisted of both residual keratin and fungal biomass. The fungal biomass was indirectly determined from the ergosterol content, which is a characteristic sterol from plasmatic membranes of fungi [35]. The ergosterol percentage from *S. aogashimaense* biomass grown in MEB for 5 days, under same fermentation conditions previously described, was employed as conversion coefficient for fungal biomass estimation. The extraction of ergosterol was done with a saponification step based on [36] and a liquid-liquid extraction step based on [33].

Thus, an accurately weighted amount of pellet mass between 10 and 100 mg was transferred to 2 mL disruption tubes containing 250 mg glass beads. Then, 0.5 mL of NaOH 4.5 M were added. The samples were mechanically disrupted in six 20 s cycles (5500 rpm with 20 s pause) and transferred to a reaction glass tube with phenolic cap. The residual material in the disruptor tube was rinsed twice with 0.5 mL of NaOH and transferred to the corresponding reaction glass tube. 1.5 mL of 1-butanol was added into each tube followed by gentle mixing using VWR test tube shaker (Galileo Equipos, Spain). All tubes were subsequently fluxed with nitrogen gas, sealed with phenolic screw caps, and heated at 85 °C for 3 h using a thermoblock device, promoting the saponification of the sample. Then, ergosterol was separated by a liquid-liquid extraction step adding 1.5 mL of methyl tert-butyl ether (MtBE) with subsequent mixing for 1 min and centrifugation at 3000 g for 5 min. The obtained upper organic phase was collected with Pasteur glass pipettes and transferred to glass test tubes. The liquid-liquid extraction with MtBE was repeated another two times. The organic phase was evaporated with a vacuum concentrator Speedvac system (Thermo Scientific, USA) at 35 °C for 1.5 h with a pressure between 5 and 10 torr. A small amount of Na_2_SO_4_ was added to remove traces of water. The dried samples were solubilized with 1 mL of acetonitrile: ethyl acetate (60:20, v/v) and vortexed for 20 s. Then, the samples were filtered with 0.22 μm PTFE hydrophobic syringe filters (SANI Membranes, Denmark) and transferred directly to 1.5 mL amber GC/HPLC vials. The vials were fluxed briefly with nitrogen gas before closing them using aluminum clip caps. A set of ergosterol standards were prepared in methanol at the following concentrations: 1000, 500, 250, 100, 50 and 10 ppm. The HPLC-UV analysis was performed using a Agilent 1260 Infinity II instrument (Agilent technologies USA) equipped with a Agilent 1260 Infinity II variable wavelength detector (Agilent technologies USA) and a C18 column Kinetex, EVO (150 × 4.6 mm, 2.6 μm particle size) (Phenomenex, USA) following the method of [6]. Two mobile phases were applied: solvent A consisting of acetonitrile, methanol and 0.1 M Tris-HCl at pH 8 (84:2:14, v/v/v), and solvent B consisting of methanol and ethyl acetate (68:32, v/v). The injection volume was 20 µL with a constant flow of 1.2 mL/min at 25 °C with the following elution gradient: from 0 to 13 min a linear change from solvent A to B; from 13 to 19 min constant elution with solvent B; from 19 to 20 min linear change from solvent B to A; and from 20 to 25 min constant elution solvent A. Detection and quantification of ergosterol was set at 280 nm.

The keratinous mass was determined by subtracting the fungal biomass from the total solids mass. The degradation efficiency was calculated as the percentage of keratinous mass loss from the initial keratin mass.

#### Ammonium content and pH of the hydrolysates

The concentration of ammonia in the hydrolysates was assessed using a Fisherbrand Accumet XL600 meter equipped with an ionic selective probe for ammonia – Orion Ammonia Gas Sensing ISE Electrode (Thermo Scientific, USA). To do so, 5 mL of hydrolysate sample were diluted with 20 mL of distilled water, and0.5 mL of ammonia ionic strength adjuster solution (Thermo Scientific, Cat. # 951211) was added. The probe was immersed in the solution while stirred with magnetic agitation and the values recorded until stable reading. The pH of the hydrolysates was measured, using a pH meter (Mettler Toledo, USA).

#### Proteolytic activity assay

Proteolytic activity of the hydrolysate samples was quantified using Sigma’s general 96-well plate proteolytic activity assay (Sigma-Aldrich, USA) with azo casein as a substrate, following protocol in [8]. A calibration curve was generated using bovine pancreas protease (Cat. No P4630) solution of known enzymatic activity calibrated following the method of Cupp-Enyar [12]. A blank with distilled water was included to correct for background absorbance. All measurements were performed in triplicates using a SPECTROstar Nano spectrophotometer (BMG Labtech, Germany), with absorbance measured at 440 nm. General proteolytic activity was chosen over keratinase activity since Keratinases are just one type of protease involved in keratin degradation. Since this process relies on a variety of enzymes, including general proteases and disulfide reductases, measuring only keratinase activity gives an incomplete picture. Assessing total protease activity better reflects the organism’s full enzymatic potential for substrate breakdown.

#### Elemental analysis and total amino acid content determination

Prior to the elemental and amino acid analysis, the samples were freeze-dried with a Labconco FreeZone2 2.5 L (LabConco, USA) and the solid fraction of the hydrolysates were analyzed. Total carbon, nitrogen and sulfur contents of the hydrolysates were determined by the Dumas method following the ISO 16,634 [19] using a Vario EL Cube elemental analyzer (Elementar Analysensysteme GmbH, Germany). The nitrogen-to-carbon ratio was calculated from these measurements, by dividing the nitrogen content by the carbon content. Amino acids were quantified on a Biochrom 30 + Amino Acid Analyzer (Biochrom Ltd., UK) following the analytical procedure described in Commission Regulation (EC) No. 152/2009. Prior to acid hydrolysis, samples were subjected to an oxidation step to enable determination of cysteine and cystine. Tryptophan was not quantified due to its susceptibility to oxidative degradation. Analyzing the amino acids present in hydrolysates obtained after the fungal hydrolysis of feathers provides valuable insights into metabolic processes and the products generated after keratin degradation, which can be utilized in various industrial applications.

#### Fourier transform infrared spectroscopy analysis (FTIR)

Hydrolysates samples were subjected to FTIR spectrometry, to do so, a total of 30 µL of samples were prepared as follows: 10 µL droplet of hydrolysate was spotted on a 384-well silicon microplate and dried at room temperature, sequentially, the same operation was repeated two times more on the same spot. FTIR spectroscopy measurements were carried out in transmission mode using a high-throughput screening extension unit (HTS-XT) coupled to a Vertex 70 FTIR spectrometer (both Bruker Optik, Germany). The FTIR system was equipped with a global mid-IR source and a deuterated L-alanine doped triglycine sulfate (DLaTGS) detector. Spectra were recorded as the ratio of the sample spectrum to the background spectrum of the empty IR transparent microplate. Each sample was measured in three technical replicates. Data acquisition and instrument control were facilitated using OPUS 8.2 software (Bruker Optik GmbH, Germany). Each of the spectra were recorded with a total of 64 scans, utilizing Blackman-Harris 3-Term apodization, a spectral resolution of 6 cm^− 1^, and a digital spacing of 1.928 cm^−1^, covering the range from 4000 to 400 cm^−1^, and employing an aperture of 6 mm.

The spectral preprocessing was performed using first rubber band baseline correction, and then extended multiplicative signal correction (EMSC) with linear and quadratic terms (EMSC) [26, 48], the region from 400 to 900 cm^− 1^ was also cut off. Following the preprocessing, the spectra were analyzed using principal component analysis (PCA), using the whole region from 4000 to 900 cm^−1^ of the preprocessed spectra. The spectral preprocessing and PCA was performed in the Orange data mining toolbox version 3.32.0 (university of Ljubjana, Slovenia) [13].

## Results and discussion

### Isolation and identification of keratinolytic fungi

**Fig. 1:**
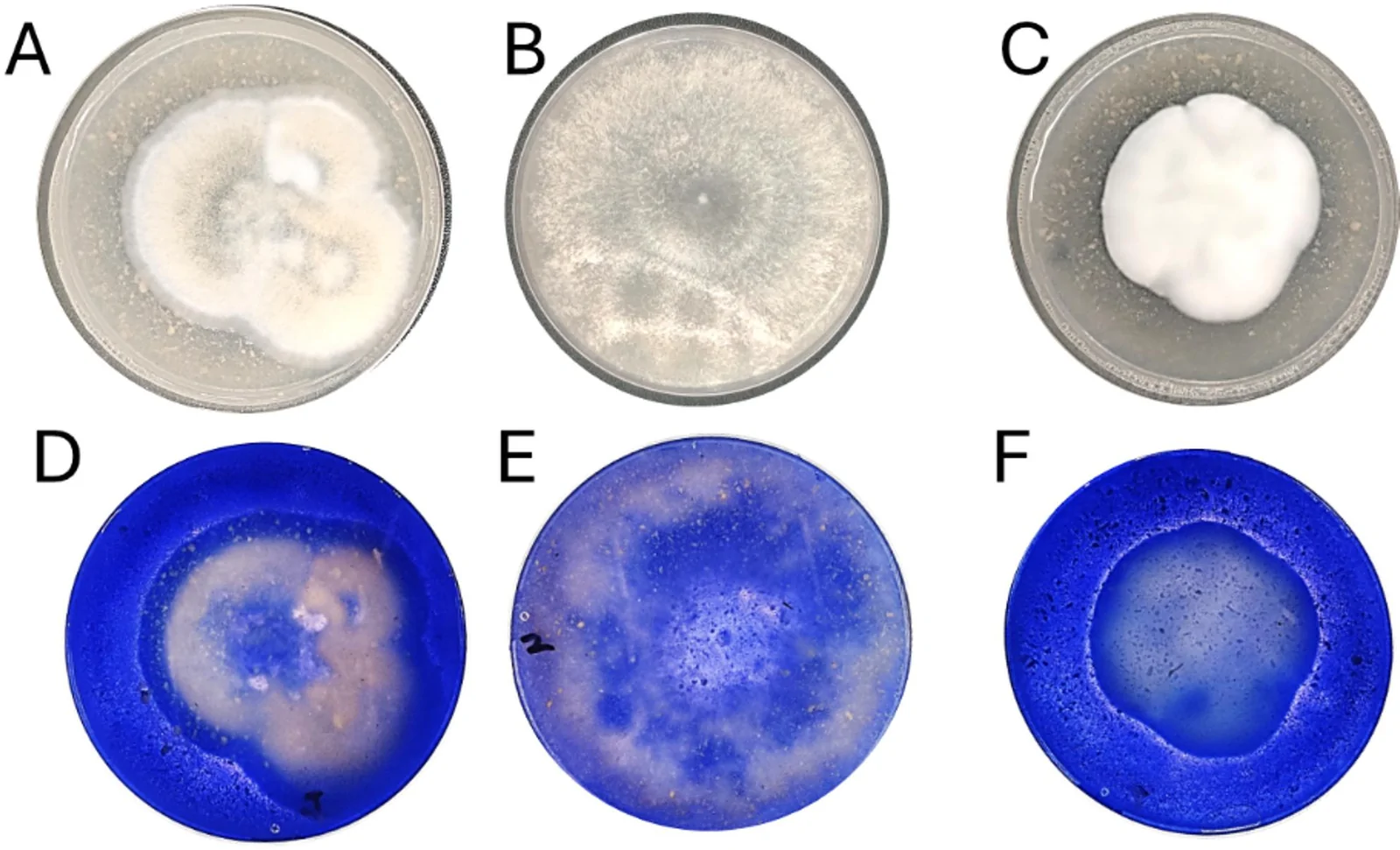
Growth of the three fungal isolates on feather meal agar (FMA). **A**–**C** show unstained colonies, and **D**–**F** show the corresponding colonies stained with Coomassie Brilliant Blue (CBB). Isolates are arranged as follows: **A** and **D**, *Aphanoascus fulvescens*; **B** and **E**, *Arthroderma uncinatum*; **C** and **F**, *Simplicillium aogashimaense*

Following inoculation of the 51 different soil suspensions onto FMA and WMA, three fungal isolates produced clear halos indicative of keratin degradation (Table 1; Fig. 1). These observations were confirmed after sub-culturing the isolates onto fresh WMA and FMA plates and staining with Coomassie Brilliant Blue (CBB) to visualize keratin hydrolysis (Fig. 1). The isolates were taxonomically identified by the Westerdijk Fungal Biodiversity Institute and assessed for biosafety, including pathogenicity potential and suitability for industrial biotechnology applications, to support the selection of candidates for further physiological and keratinolytic characterization (Table 1).

It should be noted that the isolation of microbes from field soil samples was conducted with the aim of obtaining novel keratinolytic fungal strains rather than quantify all culturable keratin-degrading microorganisms across the field experiments. Furthermore, quantifying the culturable microorganism still would not reflect the actual microbiome present in the soil sample since parts of microorganisms are not culturable [31, 52]. Consequently, the results do not necessarily reflect the effects of any specific soil treatment on the abundance of keratin-degrading microorganisms. Throughout the whole experiment, four fungi were isolated with potential keratinolytic properties, one of them was not confirmed on a new plate. All three other keratinolytic fungi were isolated from garden soils containing high levels of wool pellets, and frequently also containing eggshells and bone meal as fertilizers. While comprehensive characterization of the microbial communities in these soils was beyond the scope of this study, such analyses, particularly of keratin-degrading populations, could be addressed in future work using approaches such as metagenomic sequencing.

All three isolates produced clearing zone – halo, around the fungal colony on FMA but not on WMA, consistent with the distinct biochemical and structural requirements for microbial degradation of feather versus wool keratin [27]. On FMA, the visualization of clearing zones of fungal isolates is less clear than the one observed on bacterial isolates [43] but a halo around the colony is still visible for isolate 1 and 2 (Fig. 1D, E and F). According to the literature, none of the isolated fungi currently has established industrial application. However, from a biosafety perspective, two of the strains have previously been reported as human pathogens. *Aphanoascus fulvescensis* is described as a mild opportunistic pathogen [38] and has also been shown to degrade feathers [4]. *Arthroderma uncinatum*, a dermatophyte capable of colonizing mammalian hair, is considered a primary pathogen [50], which is consistent with its activity on keratin. However, this strain being efficient on mammalian hair, it should have been able to also degrade wool. The absence of a visible degradation halo during incubation of this fungus on wool meal agar plates likely reflects the fact that the conditions required for wool keratin degradation by this strain are not yet well characterized. In this study, wool meal agar was prepared using the same protocol as feather meal agar. However, if the optimal conditions for wool degradation differ from those for feather degradation, this approach may not be appropriate and could result in false-negative observations. Consequently, the lack of halo formation does not necessarily indicate an absence of wool-degrading capacity but rather highlights the need for condition-specific optimization. Furthermore, the strongest resilience of wool compared to feathers could explain the need for a more complex degradation system that is not achievable by the isolated strain alone.

**Table 1:** Overview of keratin-degrading fungi isolated from wool-supplemented soil and composition of the original soil samples

Fungal isolate	Soil type	Fertilizer type	Fertilizer amount (g/L)	Additionnal material	Strain identification	Reported pathogenecity	Reported keratinolytic activity
**1**	G	WP	**6**	no	*Aphanoascus fulvescens*	Mild pathogen [38]	Bohacz [4]
**2**	G	WP	**12**	P M E	*Arthroderma uncinatum*	Primary pathogen [50]	Tang et al. [50]
**3**	G	WP	**24**	M E	*Simplicillium aogashimaense*	-	-

Soil composition is indicated by the type of soil (G, garden soil), the type of wool fertilizer applied (SW, shredded wool; WP, Woolero wool pellets), and the addition of other organic amendments (P, potato peels; M, bone meal; E, eggshells)

Therefore, this study focuses on characterizing the feather-degrading properties of *S. aogashimaense*. The genus is relatively poorly characterized, with only a limited number of species described, some of which have been isolated from soil and shown to exhibit plant- or insect-associated pathogenicity [9]. For the specific isolate *S. aogashimaense*, no association with keratin degradation has been reported thus far. However, its interactions with other fungi, including antagonism against the wheat powdery mildew fungus *Blumeria graminis*, and its endophytic association with *Brachiaria brizantha*, have been documented [21, 34, 56]. This highlights the potential of *S. aogashimaense* for further agricultural applications, leveraging both its novel keratin-degradation mechanisms and its plant-beneficial traits.

### Feather degradation efficiency and proteolytic activity

**Fig. 2:**
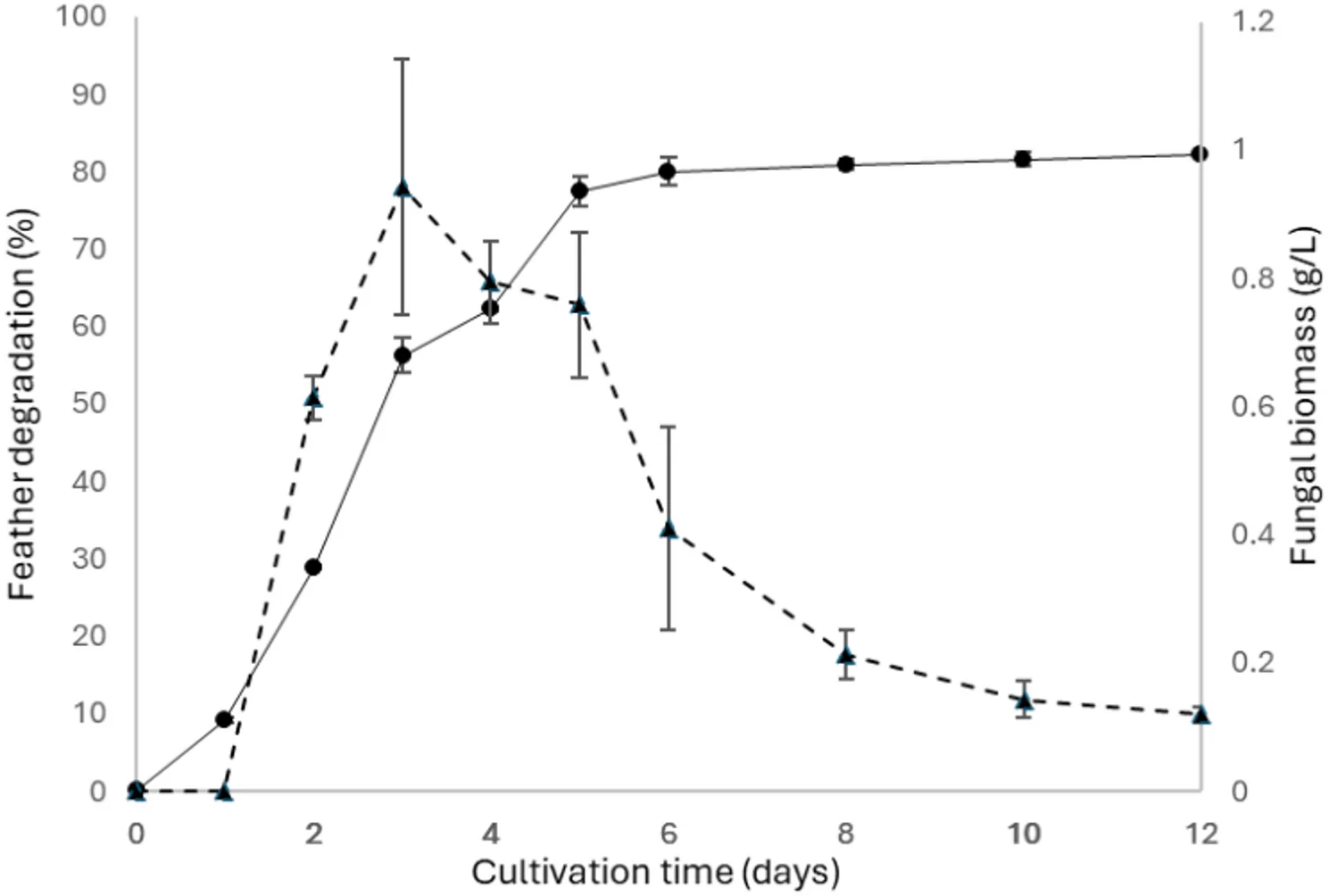
Feather degradation efficiency (solid line, dots) and fungal biomass (dashed line, triangles) during microbial hydrolysis of feather meal by *S. aogashimaense*. Error bars represent the standard deviation of biological duplicates

Both feather degradation efficiency and fungal biomass (Fig. 2) increased slowly during the first three days of hydrolysis in the shake flasks setup, with the latter one reaching 56% degradation on day 3. After this, the fungal biomass content drops slowly until the end of the experiment, while degradation efficiency keeps increasing. Indeed, from day 3 to 5, degradation continued at a moderate rate and further reached 80–82% between days 6 to 12. The continued increase in degradation efficiency, even as fungal growth slows, suggests that the fungus can access nutrients efficiently during the initial stages of hydrolysis. However, as the process progresses, due to toxic byproduct accumulation or micro-nutrients depletion, limiting further fungal growth.

The feather meal degradation rates exhibited by *S. aogashimaense* here are substantially fast and efficient. Several studies have reported lower degradation efficiencies for fungi. For example, [37] reported around 75% of degradation of feather meal using *Onygena corvina* after 5 days on incubation, using a feather meal load 5 g/L. In contrast, the degradation efficiency observed for *S. aogashimaense* was comparable to the ones reported for bacteria in our previous studies, with of 81% degradation after 7 days for *Bacillus licheniformis* and 84% after 5 days for *A. oryzae* [18, 43].

### General proteolytic activity, azocasein assay

**Fig. 3:**
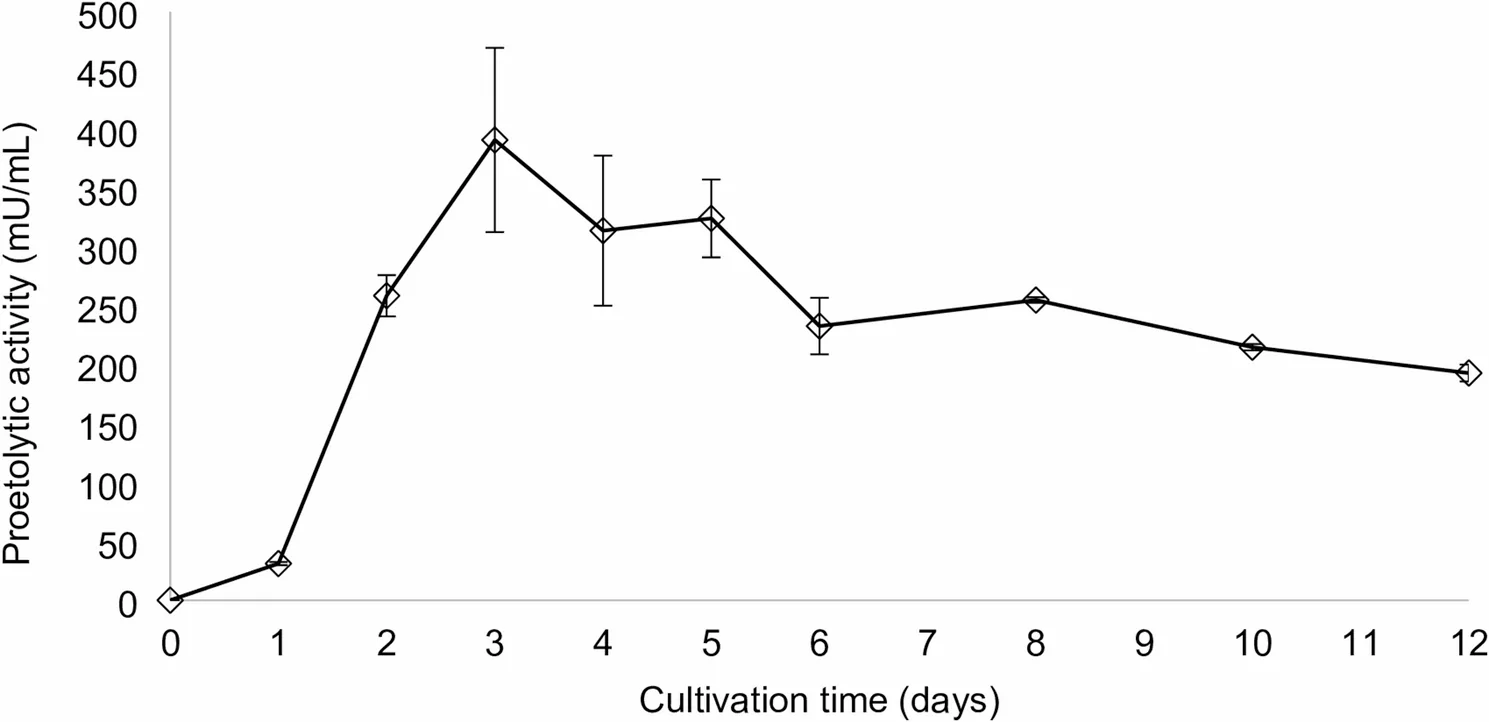
General proteolytic activity in culture supernatants (hydrolysates) during microbial hydrolysis of feather meal by *S. aogashimaense*. Error bars represent the standard deviation of two biologically independent replicates

Proteolytic activity in the hydrolysates was quantified using azocasein and is shown in Fig. 3. The activity increased sharply from day 1 to day 3, reaching a maximum of 391 mU/mL, followed by a gradual decline until day 6 and subsequent stabilization at approximately 200 mU/mL during the later stages of the experiment. In fact, this pattern mirrors closely the fungal growth along with the observed feather degradation kinetics (Fig. 2) Both fungal biomass and proteolytic activity maxima coincided at day 3, and then slowly declined.

However, it is worth noting that the magnitude of the general protease activity, a casein-based method, is not necessarily proportional to the hydrolysis efficiency, as discussed in [18]. In that publication, *Arthrobacter oryzae* and *Bacillus licheniformis* achieved similar feather degradation while differing in general protease activity values by roughly 20-fold. Such results were found to be related to differences in protease substrate specificity, which plays a crucial role in feather degradation. Since *Simplicillium aogashimaense* is a newly identified keratinolytic organism, assessing its overall proteolytic activity establishes a broader functional baseline for future studies, such as enzyme isolation and characterization. To clarify our approach, we have included a brief explanation of this rationale in both the methods and discussion sections, making our reasoning clear to readers.

### Ammonium concentration and pH levels

**Fig. 4:**
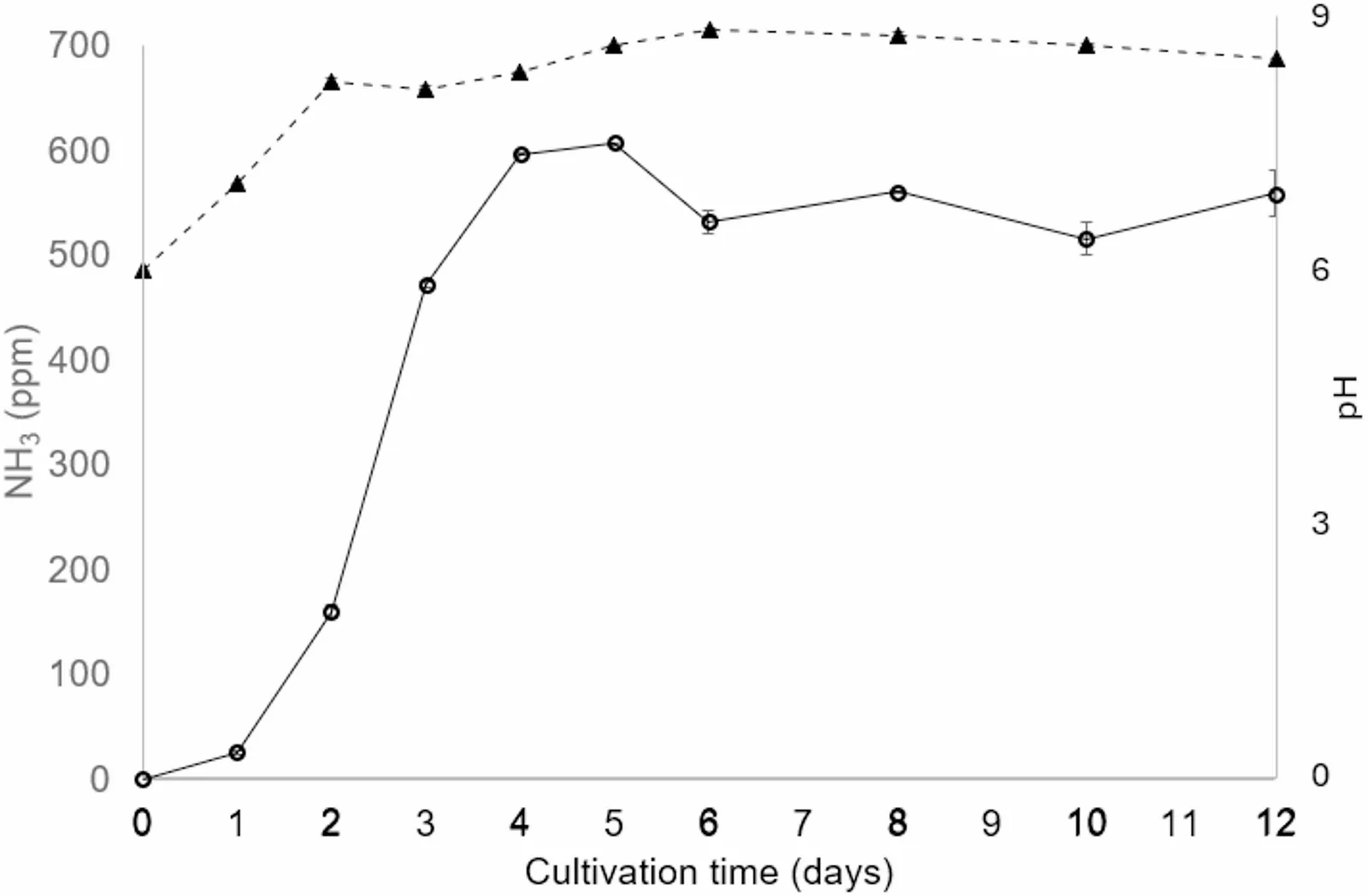
Ammonia (NH₃) concentration (solid line, dots) and pH (dashed line, triangles) in hydrolysates during feather meal hydrolysis by *S. aogashimaense*. Error bars represent the standard deviation of two biologically independent replicates

Ammonium concentrations in the hydrolysates (Fig. 4) increased sharply from day 1 to day 5, reaching a maximum of 607 ppm, followed by a slight decline until day 10. In parallel, the pH increased rapidly from an initial value of 6.0 to approximately 8.2–8.9 by day 2 and then remained relatively stable for the remaining time of the cultivation. The subsequent decrease in ammonium concentration after day 5 is likely associated with ammonia volatilization under alkaline conditions. The rapid accumulation of ammonium during the early stages of cultivation confirms that excess of nitrogen of feather meal for the fungal growth requirement, resulting in its secretion out of the cell in the form of ammonia, consequently increasing the pH. Such pH shifts from slightly acidic to alkaline values are characteristic of keratin hydrolysis and reflect combined effects of metabolic activity, enzymatic deamination, and keratin breakdown. Similar trends have been reported for both fungal and bacterial feather meal hydrolysis systems [42, 43].

### Elemental and amino acid composition of hydrolysates

**Fig. 5:**
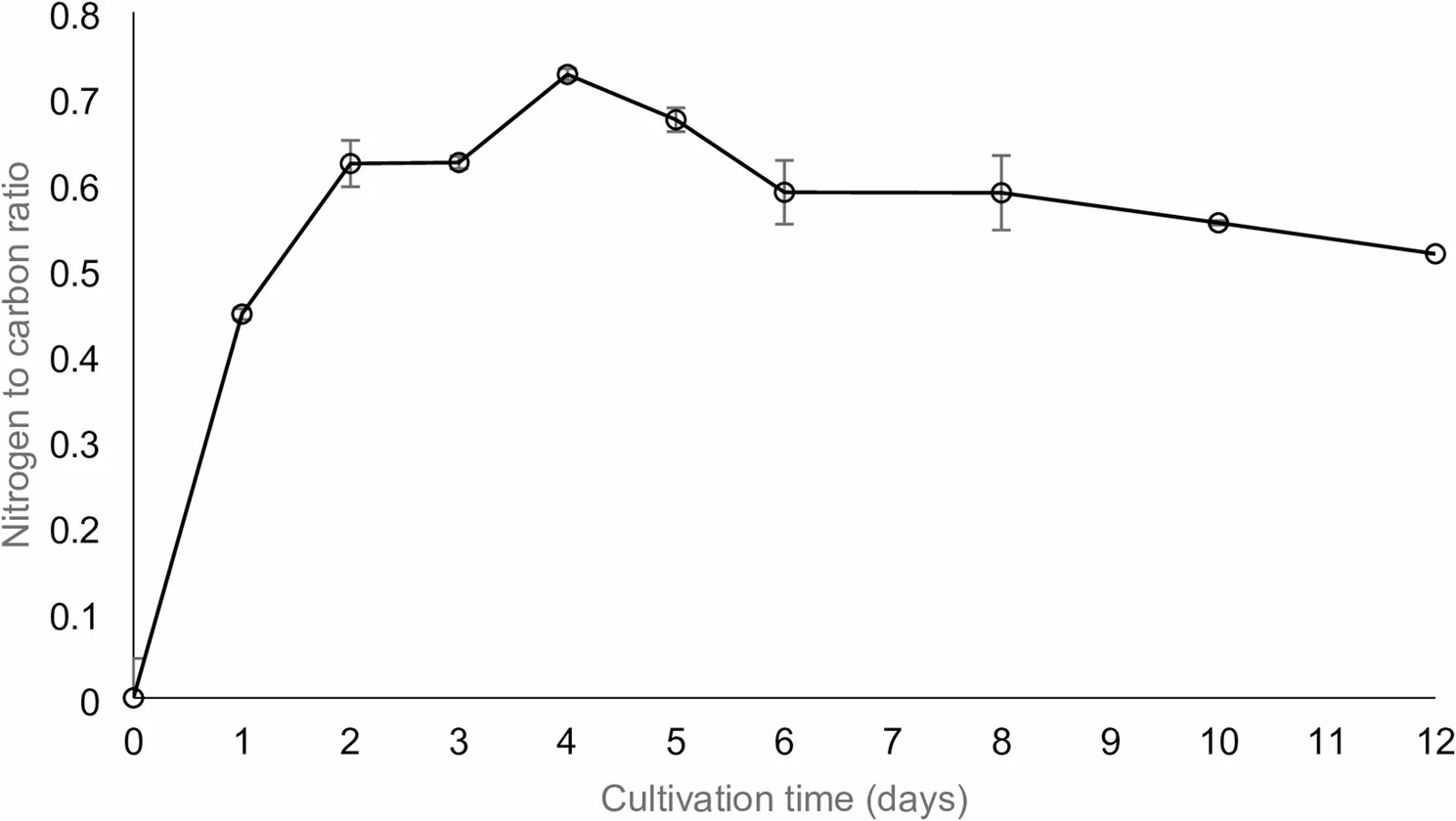
Nitrogen-to-carbon (N/C) ratio in hydrolysates during microbial hydrolysis of feather meal by *S. aogashimaense*. Values are expressed relative to the total dry matter content of each sample. Error bars represent the standard deviation of two biologically independent replicates

The nitrogen-to-carbon ratio (N/C) of the solid fraction of the hydrolysates (shown in Fig. 5, full elemental analysis provided in Supplementary Table S2) followed a pattern comparable to that of ammonia accumulation. It increased rapidly until day 4, reaching a maximum of 0.73 N/C, and then declined gradually until day 12. This suggests initial enrichment of nitrogen during early hydrolysis, followed by nitrogen relative decrease likely due to ammonia evaporation and/or metabolic consumption, as also reflected by the ammonia profile (Fig. 4). The initial increase in N/C further indicates preferential utilization of carbon relative to nitrogen during early stages of hydrolysis, consistent with the nutritional requeriments of the microbes [18, 43].

The total amino acid composition was assessed and compared with previously reported values for feather meal (FM) [18]. Changes in total amino acids throughout time are influenced by the feather hydrolysis mechanisms and preferential consumption of peptides/amino acids of the microorganism.

**Fig. 6:**
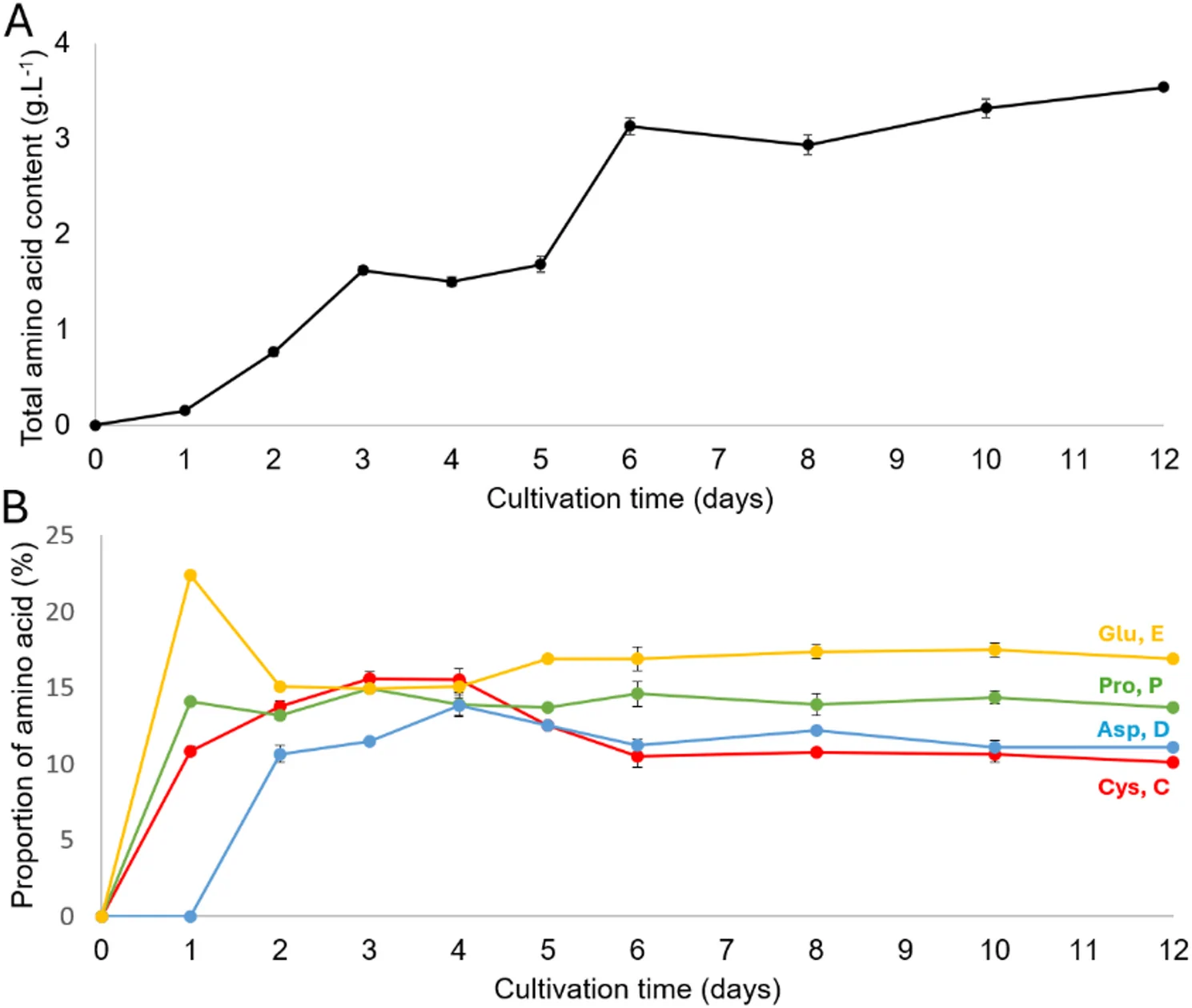
Total amino acid content (**A**) and relative abundance of selected amino acids (**B**) in hydrolysates during microbial hydrolysis of feather meal by *S. aogashimaense*. Glutamic acid (Glu, E) is shown in yellow, proline (Pro, P) in green, cysteine (Cys, C) in red, aspartic acid (Asp, D) in blue. Error bars represent the standard deviation of two biologically independent replicates. The concentration of amino acids present in the media at the start of the hydrolysis was subtracted from the measured values

Overall, total amino acid concentrations increased progressively throughout the hydrolysis period (Fig. 6A and supplementary material Table S3 and S4), following four different stages. The first stage, from the start of the experiment until day 3, a sharp increase was observed, reflecting an initial secretion of enzymes and feather meal hydrolysis. During the second stage, between day 3 and 5, total amino acid values remained relatively constant, indicating a balance between the feather hydrolysis rate and the substrate consumption rate of the fungus, which was at its maximum growth phase. During the third stage, from day 5 to 6, a pronounced increase in total amino acids was observed. This stage coincided with the death phase of the fungus and consequently with lower consumption rates, while enzymes were still actively hydrolyzing the feather meal. Finally, the fourth stage, from day 6 to 12 was characterized by a slow increase in total amino acid content, coinciding with the plateau of feather degradation, during which only the most recalcitrant feather material remained (Fig. 2).

Four amino acids: glutamic acid (Glu, E), proline (Pro, P), cysteine (Cys, C) and aspartic acid (Asp, D) were present in hydrolysates in comparatively higher concentrations than all the other amino acids, accounting together for 52 to 60% of the total amino acid content (Fig. 6B). The predominance of these amino acids over the others is expected since they are majoritarian in feather composition (supplementary material Fig S3). In comparison with bacterial feather hydrolysates [18], the same four amino acids were also present at high proportions in hydrolysates, but not as high as in the hydrolysates of this study. Each of these amino acids displayed distinct temporal dynamics.

Proline (Pro) remained at consistently high concentrations throughout the experiment. Elevated levels of proline soluble in the hydrolysates could indicate deeper hydrolysis of proline-rich regions, which are typically more resistant to proteolysis [28, 53]. However, the limited metabolic utilization of this amino acid is possibly due to the higher energetic cost of proline catabolism (Keller and Hohn [23]).

Glutamic acid (Glu) accumulated rapidly during the first day and subsequently reached a lower steady level from day 2 onward, indicating preferential utilization as an early carbon and nitrogen source. This can be expected since glutamate is a central metabolite connecting TCA cycle and biosynthesis pathways of nucleotides, amino acids and other biomolecules [11].

In contrast, aspartic acid (Asp) increased gradually until day 3 and was then consumed more slowly. Both glutamic acid and aspartic acid are part of the surface layers of keratin [16, 51], making them easily accessible in early stages of degradation.

Finally, cysteine (Cys) relative content was increasing until day 3–4 and then decreased slowly, consistent with its localization in more structurally resistant keratin [46]. Cysteine is more frequent present in the core structure of keratin and often forming disulfide bonds between them. The decrease of cysteine relative content coincided with the fungal death phase. This suggests a mechanism of disulfide bond breakdown dependent on living fungal biomass, thereby facilitating the release of cysteine-containing peptides. Upon entry into the death phase, this mechanism may be reduced, leading to a proportional decrease in cysteine release relative to other amino acids. However, the amino acid analysis used in this study included an oxidation step for cysteine and cystine (Cys-Cys) determination as one. Therefore, other analysis should be done in future to determine the mechanisms of disulfide bond breakdown.

### Biochemical profiling of hydrolysates by FTIR spectroscopy

**Fig. 7:**
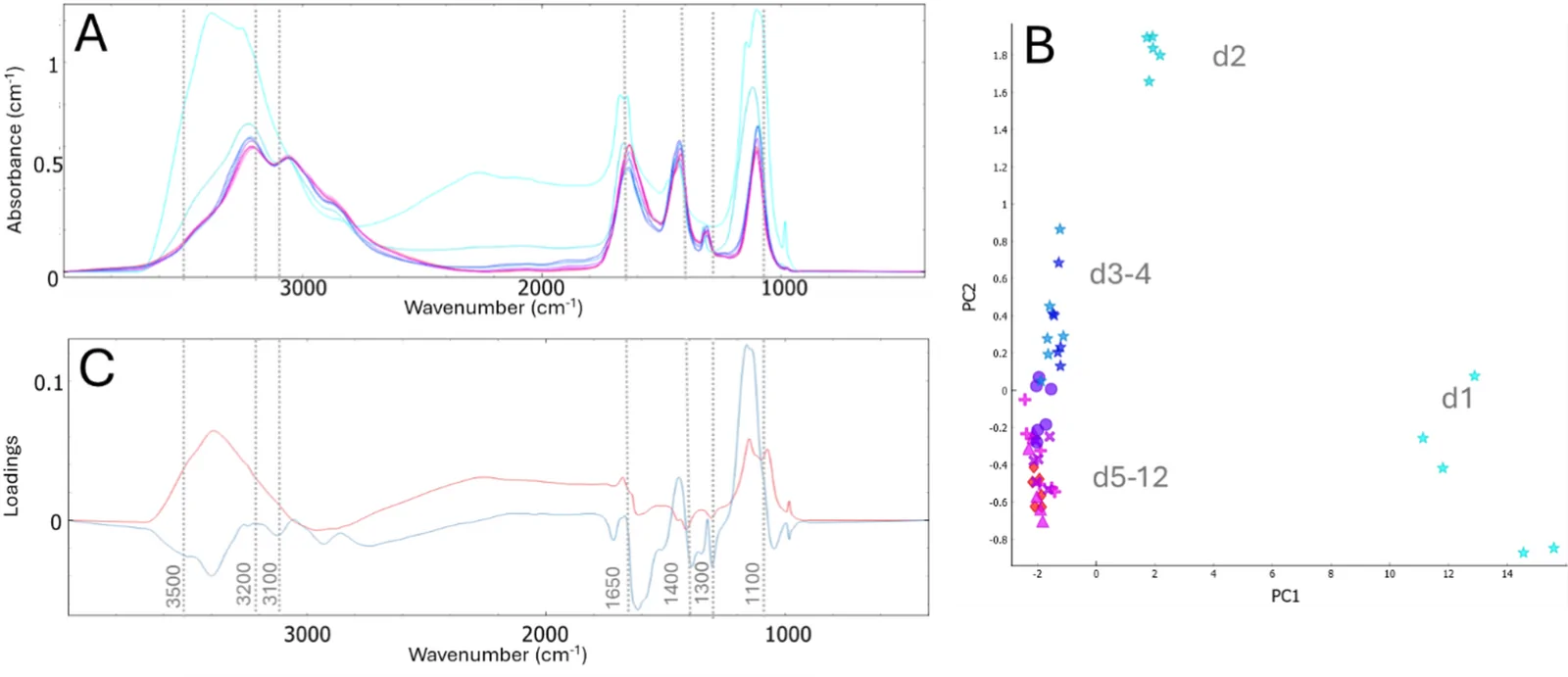
FTIR-HTS spectra, PCA scores, and loadings of hydrolysates obtained during microbial hydrolysis of feather meal by *S. aogashimaense* during 12 days of cultivation. **A** FTIR spectra, averaged across biological and technical replicates of each timepoint. Spectra are color-coded according to cultivation time, from 1 to 12, with early days in blue ad late ones in red. **B** PCA scores plot colored by cultivation time, shapes in the PCA are the following: day 1 to 4: stars, day 5: dots, day 6: x cross, day 8: triangle, day 10: cross, day 12: diamond. **C** PCA loadings for PC1 (red) and PC2 (blue). The percentage variances for the first five PCs are: 93.8, 0.23, 0.14, 0.12, and 0.05

FTIR spectroscopy enables broad characterization of molecular functional groups and overall chemical composition in complex biological samples [30, 54]. FTIR has previously been used to assess protein hydrolysis [45], and in our recent work it was used to monitor chemical changes in bacterial feather hydrolysates [18, 43, 54]. In the present study, FTIR spectroscopy was employed for the same purpose. The complete set of spectra is shown in Supplementary Fig. S2, while the main results are summarized in Fig. 7. All spectra were subjected to principal component analysis (PCA) (Fig. 7B), and the corresponding loadings are presented in Fig. 6C.

**Fig. 8:**
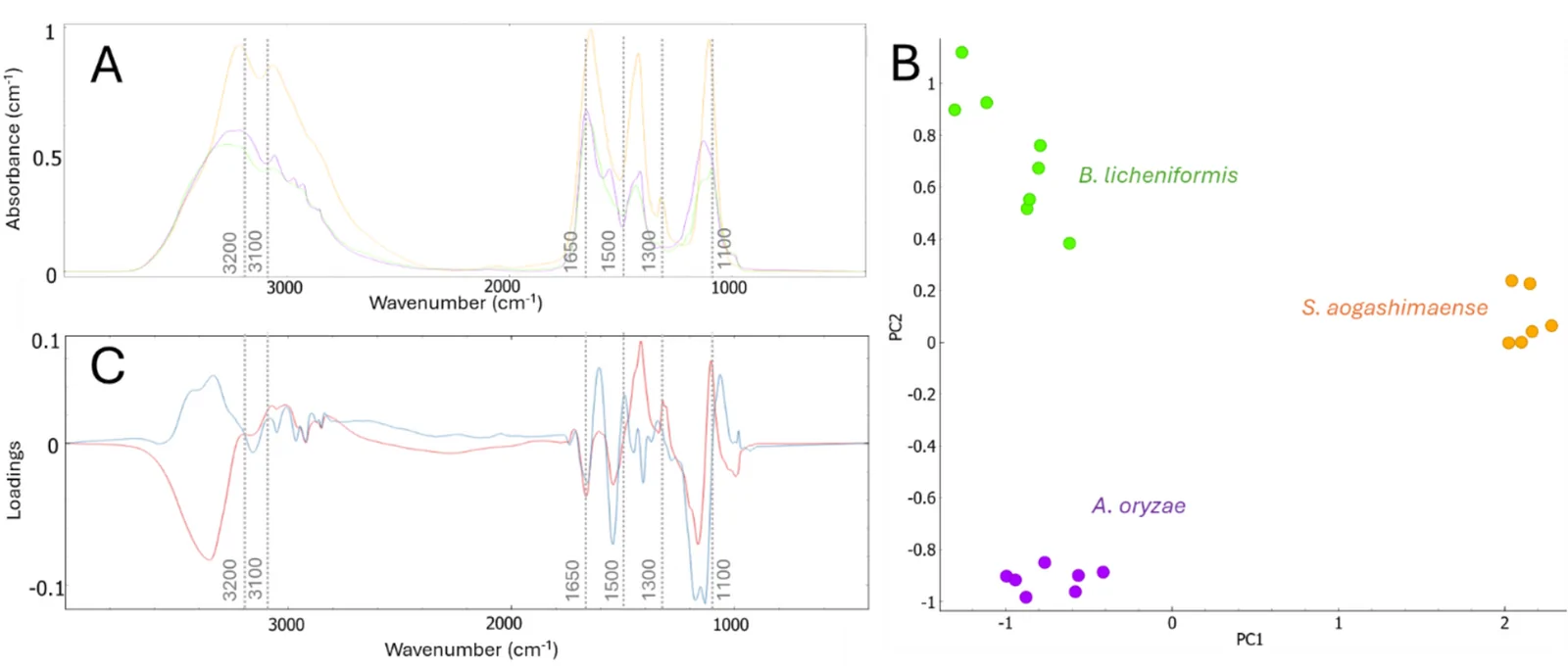
FTIR-HTS spectra, PCA scores, and loadings for feather hydrolysates obtained with *B. licheniformis* after 5 days of incubation (green), *A. oryzae* after 7 days of incubation (purple) and *S. aogashimaense* (orange). **A** FTIR spectra of hydrolysates, averaged across biological and technical replicates, colored by strain (**B**) PCA scores plot colored by strain. **C** PCA loadings for PC1 (red) and PC2 (blue). The explained variance percentages for the first five PCs are: 82.4, 16.4, 0.94, 0.31, 0.14

PCA revealed three major groups corresponding to day 1, day 2 and all subsequent sampling points. These groups were primarily separated along PC1, with additional separation along PC2. We observed that PC2 differentiated samples collected after day 3: days 3 and 4 clustered together, day 5 formed a distinct group, and days 6–12 clustered separately. This suggests that chemical changes occurring after day 6 are less pronounced than during the earlier phases of hydrolysis. Days 3 and 6 thus appear to represent key transition points, consistent with other observations in this study. Inspection of the spectra and PCA loadings identified several informative bands. These included amide A and B (N-H stretching vibrations at ~ 3300 cm^−1^ and ~ 3100 cm^−1^), O-H stretching (3500–3200 cm^−1^), aliphatic C–H vibrations (2800 cm^−1^), amide I (C = O stretching at ~ 1650 cm^−1^), amide II (1500 cm^−1^), and amide III (around 1300 cm^−1^) and finally C-O stretching vibrations (1100 cm^−1^). High positive loadings on PC1 were associated with the 3500–3200 cm⁻¹ region, associated with O–H stretching vibrations. This is consistent with the preferential consumption of carbon over nitrogen from *S. aogashimaense* during feather hydrolysis. Amino acids are de-aminated and the surplus of nitrogen is excreted in the form of ammonia. On the other hand, high positive loadings on PC2 corresponded to ~ 3200, ~3100, and ~ 2800 cm^−1^ (amide A/B and aliphatic vibrations) could be linked to differences in protein structure during intermediate stages of hydrolysis, with days 4–5 containing relatively more intact, protein-rich material than later stages (days 6–12). Positive loadings near 1650 cm^−1^ (amide I) were associated with samples from day 1 and days 6–12, indicating structured secondary protein conformations present in intact keratin at day 1 and in soluble proteins or peptides with defined secondary structure at later stages. In contrast, intermediate days were dominated by more extensively degraded peptide fragments. Amide II peaks (1500 cm^−1^) were associated with negative PC1 loadings, indicating presence of peptide bonds or soluble proteins in later-stage hydrolysates (days 3–12). Strong loadings around 1200 cm^− 1^ (amide III) on both PC1 and PC2 reflect progressive changes in protein backbone structure with increasing cultivation time. A peak around 1300 cm^−1^, likely corresponding to CH₂ deformations from aliphatic residue, was absent on day 1 but present in all later samples. Furthermore, this peak is not present in bacterial hydrolysates (Fig. 8), thus it may be due to the production of a compound by the fungus or to different degradation strategies compared to those exhibited by the bacteria. This interpretation is supported by strong negative PC2 loadings around 1100 cm^−1^ indicating polysaccharides related to maximal fungal contribution around days 2–3, followed by a decline as degradation products increasingly dominate the hydrolysate composition. Taken together, the FTIR and PCA analyses support progressive keratin degradation coupled with fungal growth throughout cultivation, characterized by cleavage of peptide bonds, disruption of keratin secondary structure, and accumulation of soluble, hydrophilic degradation products.

These results further indicate that feather meal hydrolysates generated by *S. aogashimaense* differ in their molecular composition and degradation products from those produced by bacteria [18]. The spectra of hydrolysates obtained after 6 days of hydrolysis with *S. aogashimaense* were compared with the spectra of bacterial hydrolysates from the previous study, which were obtained from the degradation of feather meal after 5 days of hydrolysis with *B. licheniformis* and 7 days with *A. oryzae.* Figure 8B shows the PCA results for the fungal and bacterial hydrolysate spectra. There is a clear distinction in PC1 between the fungal and bacterial hydrolysates, while PC2 also shows the difference between the two bacterial strains and the fungal strain. Both the fungal and bacterial hydrolysates exhibit prominent protein-related FTIR signals, including O–H stretching, amide A/B (N–H stretching), amide I and amide III bands consistent with peptide and amino acid release during keratin degradation. However, variations in amide-band intensities reflect not only differences in protein structure but also sample heterogeneity, hydration and side-chain contributions [20].

### Proposed dynamics of feather keratin degradation by S. aogashimaense

Feather meal degradation by the newly isolated *S. aogashimaense* appears to proceed via a multi-phase process, which is to be expected during microbial keratin degradation since this process relies on a cocktail of various enzymes [28]. All in all, the results presented in this study help to draw some hypothesis on the potential unfolding of this process. During the initial phase (days 1–3), degradation efficiency, proteolytic activity, and ammonium concentration increase rapidly, which could correspond to an early enzymatic attack on accessible hydrophilic keratin domains. The concomitant rise in pH reflects amino acid deamination and further supports active keratin hydrolysis. Amino acid profiling indicated potential solubilization of surface-accessible residues such as aspartic and glutamic acid, consistent with initial cleavage of unfolded and hydrophilic regions. The elevated concentrations of proline and serine in the hydrolysate most likely reflect the amino acid composition of keratin released upon enzymatic degradation. It is worth noting that serine proteases, named for the catalytic serine residue in their active site, not for any affinity towards serine-containing substrates, are well established as the principal class of microbial enzymes driving keratin breakdown Brandelli et al. [5], Sahoo [40].

This initial phase is followed by an intermediate stage (approximately days 4–6), during which degradation continues at a slower rate and general protease activity declines. This period is likely to correspond to the onset of more specialized processing of structurally resistant keratin containing hydrophobic residues. Changes in the nitrogen-to-carbon ratio further reflect this transition, with initial nitrogen enrichment driven by proteolysis followed by gradual nitrogen consumption and volatilization. Collectively, these patterns indicate a shift from intensive extracellular degradation to assimilation and intracellular metabolism.

A final phase (days 7–12) is characterized by stabilization of all measured parameters, indicating that active extracellular degradation has ceased and that the system is dominated by downstream processes such as uptake, recycling, volatilization, and chemical stabilization of soluble degradation products. FTIR analysis corroborates this progression, revealing distinct biochemical stages of keratin hydrolysis with day 3 and day 6 representing major transition points.

All in all, the ability of the studied fungal strain to degrade keratin highlights its potential for environmental applications, particularly in the sustainable management of keratin-rich wastes such as poultry feathers. By converting resistant keratin into soluble amino acids and peptides, this process offers an eco-friendly alternative to traditional disposal methods and enables the recovery of valuable biomolecules. The fungal hydrolysis approach could be integrated into waste treatment systems to reduce environmental pollution and generate feed or fertilizer ingredients, supporting both waste reduction and resource valorization.

## Conclusions

This study is the first to report evidence that *Simplicillium aogashimaense* possesses keratin-degrading capabilities. These findings expand current understanding of fungal keratin degradation and identify *S. aogashimaense* as a promising candidate for the sustainable bioconversion of feather waste and other keratin-rich residues. Future work should focus on optimizing this process and characterize the enzymatic system of *S. aogashimaense*, for example through proteomic analyses and process engineering, in order to fully unravel its biotechnological potential.

## Supplementary Information

Below is the link to the electronic supplementary material.

Supplementary Material 1.

## Data Availability

No datasets were generated or analysed during the current study.
