# Supplementary material for: First evidence of keratinolytic activity in Simplicillium aogashimaense isolated from wool-pellet–amended soil
Source: Microb Cell Fact. 2026 Jul 15;25:184. doi: 10.1186/s12934-026-03066-y (PMC13587383; doi:10.1186/s12934-026-03066-y)
Supplement: Supplementary file 1 [file 12934_2026_3066_MOESM1_ESM.docx]

***Supplementary materials***

Table S1: Composition of the tested soil samples. The type of soil is indicated as garden soil (G), compost (C), peat (P), or sand (S); wool fertilizer type as shredded wool (SW) or Woolero wool pellets (WP); and additional organic amendments as potato peels (P), bone meal (M), wine-making residue (W), or eggshells (E).

| **Sample name** | **Soil type** | **Fertilizer type** | **Fertilizer load (g/L)** | **Additional material** |
| --- | --- | --- | --- | --- |
| G1 | G | Mineral | - | - |
| G2 | G | WP | 6 | - |
| G3 | G | WP | 12 | - |
| G4 | G | WP | 24 | - |
| G5 | G | SW | 1.5 | - |
| G6 | G | SW | 3 | - |
| G7 | G | SW | 6 | - |
| G8 | G | WP | 6 | V, M, E |
| G9 | G | WP | 12 | V, M, E |
| G10 | G | WP | 24 | V, M, E |
| G11 | G | SW | 1.5 | V, M, E |
| G12 | G | SW | 3 | V, M, E |
| G13 | G | SW | 6 | V, M, E |
| G14 | G | WP | 6 | P, M, E |
| G15 | G | WP | 12 | P, M, E |
| G16 | G | WP | 24 | P, M, E |
| G17 | G | SW | 1.5 | P, M, E |
| G18 | G | SW | 3 | P, M, E |
| G19 | G | SW | 6 | P, M, E |
| G20 | G | WP | 6 | M, E |
| G21 | G | WP | 12 | M, E |
| G22 | G | WP | 24 | M, E |
| G23 | G | - | - | - |
| C1 | C | - | - | - |
| C2 | C | Mineral | - | - |
| C3 | C | WP | 6 | - |
| C4 | C | WP | 12 | - |
| C5 | C | WP | 24 | - |
| C6 | C | SW | 1.5 | - |
| C7 | C | SW | 3 | - |
| C8 | C | SW | 6 | - |
| P1 | P | - | - | - |
| P2 | P | Mineral | - | - |
| P3 | P | WP | 6 | - |
| P4 | P | WP | 12 | - |
| P5 | P | WP | 24 | - |
| P6 | P | SW | 1.5 | - |
| P7 | P | SW | 3 | - |
| P8 | P | SW | 6 | - |
| S1 | S | - | - | - |
| S2 | S | Mineral | - | - |
| S3 | S | WP | 6 | - |
| S4 | S | WP | 12 | - |
| S5 | S | WP | 24 | - |
| S6 | S | SW | 1.5 | - |
| S7 | S | SW | 3 | - |
| S8 | S | SW | 6 | - |
| Gstart | G | - | - | - |
| Cstart | C | - | - | - |
| Pstart | P | - | - | - |
| Sstart | S | - | - | - |

Table S2: Elemental composition of feather meal (FM) and hydrolysates obtained during microbial hydrolysis of feathers by *S. aogashimaense*, as determined by the Dumas method. Elemental concentrations are expressed as proportions of the total dry mass of each sample. The nitrogen-to-carbon (N/C) ratio was calculated from these values. Values represent means of biological duplicates with technical triplicates.

Table S3: Total amino acid and elemental composition of feather meal (FM) and hydrolysates obtained during microbial hydrolysis of feathers by *S. aogashimaense*. Amino acid content was calculated based on the dry mass of the hydrolysates and normalized to the total amino acid composition of each sample; values are expressed as the percentage contribution of each amino acid to the total amino acid pool. The amino acids included were: glutamic acid (Glu, E), aspartic acid (Asp, D), proline (Pro, P), arginine (Arg, R), phenylalanine (Phe, F), cysteine (Cys, C), serine (Ser, S), lysine (Lys, K), leucine (Leu, L), glycine (Gly, G), valine (Val, V), tyrosine (Tyr, Y), threonine (Thr, T), isoleucine (Ile, I), histidine (His, H) and methionine (Met, M). values represent means of biological duplicates with technical triplicates.

Table S4: Amino acid profile feather meal (FM), and hydrolysates obtained from the microbial hydrolysis of feather meal by *S. aogashimaense*. With Glutamic acid (Glu, E), Aspartic acid (Asp, D), Proline (Pro, P), Arginine (Arg, R), Phenylalanine (Phe, F), Cysteine (Cys, C), Serine (Ser, S), Lysine (Lys, K), Leucine (Leu, L), Glycine (Gly, G), Valine (Val, V), Tyrosine (Tyr, Y), Threonine (Thr, T), Isoleucine (Ile, I), Histidine (His, H), and Methionine (Met, M)

Keratin mass in the pellet	and soluble solid in the hydrolysates (g/L)

| Clutivation time (day) | Keratin mass (g/L) | Hydrolysate soluble solids (g/L) |
| --- | --- | --- |
| 0 | 10 | 2.24 |
| 1 | 9.078 | 1.8798 |
| 2 | 7.10751 | 2.95996 |
| 3 | 4.37197 | 6.10212 |
| 4 | 3.77702 | 6.19072 |
| 5 | 2.2554 | 6.86951 |
| 6 | 1.99856 | 8.99276 |
| 8 | 1.91032 | 8.80588 |
| 10 | 1.84014 | 9.21739 |
| 12 | 1.78891 | 9.18973 |

Figure S1: Soluble fraction of solid in the hydrolysates (blue) and keratinous mass in the pellet (green) obtained during microbial hydrolysis of feathers by *S. aogashimaense*. Error bars represent the standard deviation of biological duplicates.

Figure S2: FTIR-HTS spectra of fungal feather hydrolysates. Shown are FTIR spectra of hydrolysates obtained during microbial hydrolysis of feather meal by *S. aogashimaense*. Spectra are color-coded by cultivation time from 1 to 12, with early days in blue and later ones in red.
